# The epidemiology of bacterial vaginosis in relation to sexual behaviour

**DOI:** 10.1186/1471-2334-10-81

**Published:** 2010-03-30

**Authors:** Hans Verstraelen, Rita Verhelst, Mario Vaneechoutte, Marleen Temmerman

**Affiliations:** 1 Department of Obstetrics & Gynaecology, Faculty of Medicine and Health Sciences, Ghent University, Ghent, Belgium; 2 Laboratory Bacteriology Research, Department of Clinical Chemistry, Microbiology, and Immunology, Faculty of Medicine and Health Sciences, Ghent University, Ghent, Belgium

## Abstract

**Background:**

Bacterial vaginosis (BV) has been most consistently linked to sexual behaviour, and the epidemiological profile of BV mirrors that of established sexually transmitted infections (STIs). It remains a matter of debate however whether BV pathogenesis does actually involve sexual transmission of pathogenic micro-organisms from men to women. We therefore made a critical appraisal of the literature on BV in relation to sexual behaviour.

**Discussion:**

*G. vaginalis *carriage and BV occurs rarely with children, but has been observed among adolescent, even sexually non-experienced girls, contradicting that sexual transmission is a necessary prerequisite to disease acquisition. *G. vaginalis *carriage is enhanced by penetrative sexual contact but also by non-penetrative digito-genital contact and oral sex, again indicating that sex *per se*, but not necessarily coital transmission is involved. Several observations also point at female-to-male rather than at male-to-female transmission of *G. vaginalis*, presumably explaining the high concordance rates of *G. vaginalis *carriage among couples. Male antibiotic treatment has not been found to protect against BV, condom use is slightly protective, whereas male circumcision might protect against BV. BV is also common among women-who-have-sex-with-women and this relates at least in part to non-coital sexual behaviours. Though male-to-female transmission cannot be ruled out, overall there is little evidence that BV acts as an STD. Rather, we suggest BV may be considered a sexually enhanced disease (SED), with frequency of intercourse being a critical factor. This may relate to two distinct pathogenetic mechanisms: (1) in case of unprotected intercourse alkalinisation of the vaginal niche enhances a shift from lactobacilli-dominated microflora to a BV-like type of microflora and (2) in case of unprotected and protected intercourse mechanical transfer of perineal enteric bacteria is enhanced by coitus. A similar mechanism of mechanical transfer may explain the consistent link between non-coital sexual acts and BV. Similar observations supporting the SED pathogenetic model have been made for vaginal candidiasis and for urinary tract infection.

**Summary:**

Though male-to-female transmission cannot be ruled out, overall there is incomplete evidence that BV acts as an STI. We believe however that BV may be considered a *sexually enhanced disease*, with frequency of intercourse being a critical factor.

## Background

### Bacterial vaginosis: a brief introduction

Bacterial vaginosis (BV) is a condition characterised by the partial loss of the indigenous vaginal lactobacilli on the one hand, and polymicrobial anaerobic overgrowth of the vaginal mucosa on the other hand [1]. Although BV remains often asymptomatic, it still is, along with vulvovaginal candidiasis, the most common cause of vaginitis, and hence among the commonest reasons for women to seek medical help [2]. In recent years BV has further emerged as a global issue of concern due to its association with ascending genital tract infection and with sexually transmitted infections [3]. Infections related to BV may broadly be categorized as opportunistic infections with BV-associated bacteria and as infections due to sexually transmitted agents. In the first category, ascending genital tract infection with BV-related pathogens has been associated with postabortion [4] and postpartum endometritis [5], pelvic inflammatory disease (PID) [6,7], and, during pregnancy, late foetal loss and spontaneous preterm birth [8,9]. In the second category, BV renders women particularly vulnerable to the acquisition of *Trichomonas vaginalis*, *Neisseria gonorrhoeae *[10,11], *Chlamydia trachomatis *[11], HSV-2 [12] and HIV-1 [13-15]. Moreover, it has been documented that bacterial vaginosis propagates viral replication and vaginal shedding of the HIV-1 [16-18] and HSV-2 [19] viruses, thereby further enhancing the spread of these viruses.

Failure to control the high prevalence of BV has therefore now become a global issue of concern. As standard treatment with antibiotics tends to be ineffective in the long run [20], more efforts may be directed to preventive measures to reduce the incidence of BV. In particular, among the most prominent risk factors, sexual behaviour and vaginal douching are modifiable risk factors, e.g. a recent douching cessation trial documented a significant reduction in BV incidence following a douching cessation intervention [21]. Sex education may also add to the prevention of BV, provided that we have sufficient knowledge as to how sexual behaviour exactly enhances BV acquisition. Understanding the contribution of sexual activity to BV pathogenesis is therefore integral to improve the management and prevention of BV and to reduce its associated complications.

### Bacterial vaginosis and sexual behaviour

Of all risk factors explored thus far, sexual behaviour-related characteristics have been most consistently associated with BV. This extensive body of literature is at first sight not entirely consistent however, as BV has been associated with a variety of sexual behaviour-related characteristics including young age at coitarche, life time number of sex partners, a recent history of multiple sex partners, and a recent history of a new sex partner. These inconsistencies make it difficult to define what genuinely represents high-risk sexual behaviour to the acquisition of BV. Possibly, different studies may have been better designed to identify certain epidemiological measures than others, while the aforementioned measures of sexual behaviour may also be interdependent. Moreover, some risk factors identified may be proxy variables to the true risk exposure, e.g. a new sex partner might be predictive to frequency of intercourse. If anything, the aforementioned risk factors all seem to point somehow at a consistent association between level of sexual activity and the odds of acquiring of BV. It remains a matter of debate however whether BV pathogenesis does actually involve sexual transmission of pathogenic micro-organisms from men to women [22].

We therefore sought to review the literature on BV epidemiology in relation to sexual behaviour thereby emphasizing on those data that could provide insight into the disease mechanisms in relation to sexual behaviour. In particular, we reviewed the data on the transmissibility of BV and the indications pro an contra transmission of BV from male to female, from female to male, from female to female, and further addressed the association between non-coital sexual behaviours in relation to BV and the occurrence of BV in sexually inexperienced women.

It may be acknowledged that the literature on BV epidemiology has largely focused on the presence of *G. vaginalis*, which is a key pathogen to BV, also numerically dominant in the biofilm mode of overgrowth in BV, but not necessarily the BV initiating or causative agent. Throughout this review we also discuss these data on *G. vaginalis*, though we recognize that the patterns of sexual transmission for G. vaginalis may not be the same as those for BV.

## Discussion

### Observations on the transmissibility of bv

Early experiments conducted in the 1950s and 1960s are the only studies thus far that addressed the transmissibility of BV in a direct manner. In particular, Gardner and Dukes [23] accomplished to induce BV in 11 of 15 female volunteers who were inoculated directly with vaginal secretions from patients with BV. Conversely, when they inoculated women with pure culture *Gardnerella vaginalis*, BV occurred in only one in 13 volunteering women. However, Criswell *et al *[24], did manage to induce BV in 7 out of 29 volunteering women when using higher inocula of pure culture *Gardnerella*. Hence, in these early experiments, the transmissible nature of BV had been clearly demonstrated.

The contention of BV as a transmissible disease then gained even more significance as epidemiological data pointed at concurrent carriage of *G. vaginalis *by women with BV and their male partners. Gardner and Dukes isolated *G. vaginalis *from the urethra in 45 of 47 male partners of women with BV and later on, Pheifer *et al *detected *G. vaginalis *in the urethra of 27 of 34 partners of BV patients [23,25]. Most recently, Swidsinski *et al *assessed *G. vaginalis *carriage in a large cross-sectional study [26] involving different population groups. Building further on the discovery of the BV biofilm [27,28], Swidsinski *et al *first described that the presence of *G. vaginalis *can also reliably be assessed through fluorescence-in-situ-hybridisation (FISH) analysis on desquamated epithelial cells in urine in both women and men. In this study, the presence of *G. vaginalis *was further referred to as "dispersed" *Gardnerella*, consisting of loosely dispersed *Gardnerella *cells, and as "cohesive" *Gardnerella*, consisting of clustered *Gardnerella *cells adhesive to the epithelium, the latter indicative of the presence of the *Gardnerella *biofilm, previously shown an obligate finding in BV [27]. The authors enrolled among others, 20 women with symptomatic BV and 10 of their partners and 72 consecutive married pregnant women and their 72 partners. It was shown that the 20 women with symptomatic BV consistently presented with cohesive *Gardnerella *as did the 10 partners investigated. Among the 72 married pregnant women and their 72 partners, dispersed *Gardnerella *was found in 14% and cohesive *Gardnerella *in 17% of the females. Again, cohesive *Gardnerella *was consistently found among the partners of the women with cohesive *Gardnerella*, for whom the samples were analysable. No such concordance was observed for dispersed *Gardnerella*. Hence, the previously known strong concordance of *G. vaginalis *carriage by both partners when a woman has BV was herewith confirmed but further refined by the almost absolute concordance of cohesive *Gardnerella *carriage, which might indicate that this biofilm mode of growth represents the infectious mode of *Gardnerella *and/or BV.

Following the demonstration of transmissibility of G. vaginalis isolated from pure culture, the recognition of male carriage, and the concordance of *G. vaginalis *carriage between couples, Piot *et al *provided further evidence of sexual transmission by obtaining vaginal cultures from 12 women with BV and urethral cultures from their 12 male consorts within 24 hours [29]. The *G. vaginalis *biotypes isolated from both partners were the same for 11 of the couples (p = 0.005), strongly suggestive for sexual transmission of *G. vaginalis*.

### Observations that support male-to-female heterosexual transmission

Since male *G. vaginalis *carriage was first demonstrated, as mentioned above, among male partners of patients with BV, it has long been postulated that male carriage might indicate the presence of a male reservoir possibly leading to male-to-female transmission, although this assumption is not unequivocally supported by the literature. We herewith review the data on male carriage of *G. vaginalis *and on measures directed towards prevention of male-to-female transmission, including partner treatment with antibiotics, condom use and male circumcision.

The two largest cohort studies on male carriage of *G. vaginalis *conducted until present - both involving male attendees of a sexually transmitted disease clinic - documented male urethral carriage of *G. vaginalis *at a rate of 11.4% (49/430) in the UK [30] and of 4.5% (10/309) in Sweden [31]. As a matter of fact, male carriage of *G. vaginalis *may even be higher than estimated from the aforementioned studies, as urethral sampling may not be the optimal approach to document it. Kinghorn *et al *found a significantly higher rate of *G. vaginalis *isolation from preputial than from urethral swabs [32]. Swidsinski *et al *recently made a similar observation, and found that desquamated epithelial cells loaded with *G. vaginalis *could only reliably be recovered in a urine specimen if the praeputium was not pulled back during voiding [26]. In this manner, it was shown that among 100 men admitted to a department of internal medicine, dispersed *Gardnerella *occurred in 4% of males, while cohesive *Gardnerella *was present in 7% [26]. In addition, several culture-based studies have also documented the presence of *G. vaginalis *in semen samples [33-38] - possibly pointing at a seminal or prostatic reservoir - whereby in one study *G. vaginalis *was recovered from semen in as much as 38% of 58 men attending an infertility clinic [34]. Intriguingly, preceding the renowned description of *G. vaginalis *as the causative agent in non-specific vaginitis by Gardner and Dukes [23], the very first description of the species now known as *G. vaginalis *was in association with prostatitis [39].

Up to date, six randomized controlled trials [40-45] have addressed the effectiveness of male partner treatment in the treatment of BV. Five out of the six studies failed to document any benefit from male partner treatment with antibiotics [40-42,44,45]. It may be acknowledged here, that most of these studies suffer from multiple methodological shortcomings [46] including small sample sizes and large drop out rates. Moreover, the antibiotic regimens applied to male partners of women diagnosed with BV are mostly single doses or short courses with metronidazole or tinidazole, i.e. treatments that have been documented to be also poorly effective in women with BV and that are therefore not recommended by the CDC [47]. In only one of the six RCTs, a CDC-recommended regimen for women was administered to the spouses of women with BV, consisting of a 7-day course of oral clindamycin [45], though again without any noticeable effect. Finally, what one really wants to know is whether male treatment might prevent the recurrence of BV among their female partners, presuming that women might get re-infected from a male reservoir. This was addressed in two studies with a 3 month follow-up [42,45] whereby a recurrence rate of at least 50% among women is expected [48]. Both these studies [42,45] failed to document any benefit of male sexual partner treatment on 3 month cure rates among their female partners.

It may be concluded that the evidence suggests that there is no benefit, i.e. reduction of BV occurrence in women, by treating the sexual partners of women with BV with the drug regimens tested [46]. It may further be acknowledged that, when assuming a male-to-female route of transmission, the true effect of male treatment on the incidence of BV might at best be evaluated in a study in which male carriers would be treated prophylactically. Also, direct assessment of efficacy of antibiotic treatment for eradication of *G. vaginalis *and other BV-associated micro-organisms in male partners would be essential, as the lack of effect of male treatment on recurrence of BV in female partners not necessarily excludes male-to-female reinfection, since male treatment may not eradicate biofilm-associated *G. vaginalis*.

With regard to condom use as a means of preventing BV, six cross-sectional studies [49-54] yielded contradicting results. Longitudinal and cohort studies on the other hand are more in line with each other towards a beneficial effect of condom use *vis-à-vis *BV acquisition [55-58], although the overall observed effect tends to be rather limited with an average relative risk reduction associated with condom use estimated at merely 20% in a recent meta-analysis [59]. The two most recent studies also addressed recurrent BV [58,60]. Hutchinson *et al *found a very strong overall protective effect of consistent condom use on the occurrence of both incident and recurrent BV in a three-year follow-up study (adjusted odds ratio 0.37, 95% CI 0.20-0.70) [58]. Yotebieng *et al *on the other hand found that consistent condom use in a 6-month follow-up study was protective against incident BV, but not against recurrent BV [60]. Hence, the evidence on consistent condom use as a protective means for BV, overall, seems to suggest a rather moderate effect in the prevention of BV.

If the preputial space is suspected to act as the male reservoir of BV-associated micro-organisms - as was first suggested by Kinghorn *et al *[32] and recently reinforced by the findings by Swidsinski *et al *[26], who found more *G. vaginalis *in men voiding without retracting the praeputium, then male circumcision is expected to have a protective effect on the occurrence of BV. However, results from a limited number of studies are contradictive again. In a US retrospective case-control study no difference was found in the prevalence of BV between women with circumcised partners compared to those with uncircumcised partners [61]. In a large, very well-designed, randomised controlled trial involving a large cohort of young men in Rakai, Uganda however, male circumcision was associated with a significantly decreased risk (adjusted prevalence risk ratio 0.60, 95% CI 0.38-0.94) of BV at one year follow-up [62].

### Observations that support female-to-male heterosexual transmission

One of the earliest observations casting doubt over the purportedly male-to-female sexual transmission route comes from a large cohort study on urethral carriage of *G. vaginalis *among men [30]. In this study, Dawson *et al *isolated *G. vaginalis *from 11.4% of urethral samples from 430 consecutive male patients attending a clinic for sexually transmitted disease [30]. Interestingly, the authors documented that the recovery rate of urethral *G. vaginalis *was significantly higher in heterosexual men (14.5%) than among homosexual men (4.5%) (p < 0.001). This finding suggests that, somehow, heterosexual contact enhances male carriage of *G. vaginalis*, as if female-to-male transmission rather than the opposite route is at stake. Ten years later, Holst conducted an elegant experiment that corroborates this hypothesis [63]. Holst first documented that several BV-associated organisms, including *Mobiluncus mulieris*, *M. curtisii *and *G. vaginalis*, could be isolated from the urethras and/or coronal sulci of 10 in 44 male consorts (22.7%) of women with BV. However, after two weeks of consistent condom use during regular sexual intercourse, these BV index species disappeared from all men [63], suggesting that transient carriage of BV-associated micro-organisms by men actually may ensue from (continued) female-to-male contamination during intercourse. A very recent study by Schwebke *et al *[64] corroborates this. In this study, Schwebke *et al *[64] investigated male carriage of *G. vaginalis *through species-specific PCR in urine, on a urethral swab, and on a swab obtained from the coronal sulcus from 47 men, of whom 23 were partners of women with BV and 24 were partners of women without BV. Overall, *G. vaginalis *was detected in 12 males. The authors observed that the 11 participants who did use a condom at the last sexual encounter were all negative for *G. vaginalis*, whereas one in three men (12/36) who did not use a condom at the last sexual encounter was positive for *Gardnerella*. Moreover, none of the 5 participants who stated that they always used a condom for the past 3 months had *G. vaginalis*, whereas 12/42 of those who did not always use a condom in the past 3 months were positive for *G. vaginalis *[64]. Thus, Schwebke *et al *found that *G. vaginalis *carriage by men is closely linked to condom use, and hence this is suggestive for a female-to-male transmission-like route of infection, as had been suggested by Holst [63] two decades ago.

These findings may at least in part explain the high concordance rates between women with BV and their male consorts as mentioned above and may further explain the low success rate of BV eradication in women by condom use by their partners [59].

In addition, a female-to-male route of transmission may further be consistent with the observation that *G. vaginalis *is not present in prepubertal boys, as apparent from one study involving 99 boys with negative cultures for *G. vaginalis *from the urethra, glans, and rectum [65]. In accordance, in a study comprising 50 adolescent males who had not engaged yet in sexual activity/who were sexually inexperienced, *G. vaginalis *was isolated from the urethra in only one [66] and in a study by the same authors, involving 50 recently married, young men in monogamous relationships with no history of STDs, *G. vaginalis *was isolated from none of them [66].

### Observations on bacterial vagiginosis resulting from non-coital sexual acts in heterosexuals

Among heterosexual women, non-coital sexual behaviours, including receptive oral sex, receptive anal sex, and non-penetrative digito-genital contact have also been identified to confer an increased risk of BV acquisition.

Nandwani *et al *and Tchamouroff and Panja reported on a prospective cohort study, including 256 heterosexual female patients, attending a genito-urinary medicine clinic, and found a highly significant difference in the rate of BV among women who reported receptive oral sex in the previous four weeks (41/111 or 37%) as compared to women who did not experience cunnilingus in the past four weeks (14/145 or 10%, p < 0.001) [67,68]. Schwebke *et al *also found that an unstable microflora - as defined by the number of episodes of vaginal microflora shifting away from a *Lactobacillus*-dominated microbiota to a bacterial vaginosis-like profile - was significantly associated with more frequent episodes of receptive oral sex [69]. Interestingly, a similar observation has been repeatedly made with regard to cunnilingus and vaginal *Candida *infection [70,71,54] and this was not explained by differences in extragenital carriage of *Candida *by the women nor by differences in extragenital and genital carriage of *Candida *by their partners [70].

Unprotected receptive anal sex [72,73] and unprotected receptive anal sex before vaginal intercourse [74] have also been associated with BV.

In a study involving 44 self-reported virginal women, of which 27 provided detailed information regarding sexual practices by a self-administered questionnaire, self-collected tampons were tested for *G. vaginalis *and *A. vaginae *through species-specific PCRs [75]. Surprisingly, it was found that *G. vaginalis *carriage in these virginal women was very strongly associated with oral sex and non-penetrative digito-genital contact [75]. Similarly, Fethers *et al *very recently documented an association among 17-21-year-old females between noncoital sexual practices (oral sex and non-penetrative digito-genital contact) and the occurrence of BV [76].

Similarly, recurrent vaginal candidiasis has also been associated with a history of recent masturbating with saliva by both the patient as her partner, while this was not explained by oral *Candida *carriage [70]. Recurrent urinary tract infection has also been associated with frequent masturbation [77].

### Observations on the occurrence of bacterial vaginosis in sexually inexperienced women

Clearly, there is ample evidence that BV is not confined to women who are or were ever engaged in a heterosexual relationship, much in contrast to traditional STDs, indicating that heterosexual penetrative contact is definitely not a necessary prerequisite to the acquisition of BV.

Swidsinski *et al *investigated *G. vaginalis *carriage in 50 premenarchal girls and found that, dispersed *Gardnerella *occurred in 10%, whereas none of the 50 girls showed cohesive, i.e. biofilm-associated, *Gardnerella *[26]. It has to be acknowledged here, that higher prevalence rates of *G. vaginalis *have been found in sexually abused girls as compared to non-abused girls in most [78-80] though not all studies [81]. Apart from *G. vaginalis *carriage, bacterial vaginosis in children is however rare. Anecdotically, Papanikolaou *et al *reported one case of recurrent BV in a 17-year old adolescent with an intact hymen [82].

In contrast, once beyond the menarche, BV also occurs among sexually inexperienced adolescents and virginal women according to several studies [83-85], albeit at lower rates on average as compared to sexually active reproductive aged-women. This contention was recently challenged however by an Australian study comprising 528 young women of which 25 women had BV [76] The largest sample up to date involved US women, entering the military, with a mean age of 19.1 years. In this study, BV in women reporting to never have had vaginal intercourse occurred at a rate of 18%, whereas their sexually experienced counterparts had BV at a rate of 28% [84]. The findings on BV prevalence in the large cohort of US women entering the military are in line with those on *G. vaginalis *epidemiology with a limited number of studies also documenting higher rates of *G. vaginalis *carriage among sexually active adolescents. Shafer *et al *reported that up to one third of non-sexually active adolescents harboured *G. vaginalis*, which was significantly less than in sexually active adolescents (60%) [86]. Another culture-based study of 120 asymptomatic 14- to 17-year-old females found a twofold lower prevalence of *G. vaginalis *(17%) in women who reported no penetrative sexual activity compared with sexually active females (34%), albeit a non-statistically significant difference [87]. So, overall, several lines of evidence corroborate that the BV incidence is increased by sexual activity, but clearly also contradict exclusive heterosexual transmission.

### Observations that support female-to-female transmission

Women with lesbian orientation or generally women-who-have-sex-with-women (WSW) not only present rather consistently with very high rates of BV, but monogamous lesbian couples also present with almost absolute concordance rates of vaginal microflora characteristics in terms of presence of lactobacilli in general and of hydrogen peroxide-producing lactobacilli in particular, in terms of presence of BV -associated organisms [88]. These observations strongly suggest female-to-female between-partner transmission of BV. These observations may indicate strong between partner transmission or may just as well point to the higher occurrence among lesbian women of certain BV-enhancing sexual practices (*see below*). Indeed, lesbian women have been shown to be generally monogamous, to have low promiscuity and to have an overall low incidence of STDs [89]. Although many lesbian women do have a history of heterosexual contact, this has not been an explanatory factor to the occurrence of BV [89]. Among the common exposures investigated thus far, the very strong association between same gender sex and BV is apparently not explained by confounders such as douching, though to some extent by higher rates of smoking [89-91]. As to sexual behaviour-related characteristics among WSW, an increased frequency of sexual activity does not seem to be associated with the prevalence of BV whereas several studies found an association with increasing numbers of lifetime sexual female partners [89-91], similar to what is observed with heterosexual women. From a very detailed analysis including a number of self-reported sexual acts however, Marrazzo *et al *retained significant associations between BV risk among lesbians with a lack to always clean an insertive sex toy before use, with oral-anal sex, and with recent genital-genital contact with a female partner [88]. Albeit based on a small series of patients, Tchamouroff and Panja also suggested a link between receptive oral sex and BV among WSW [68].

### Comment

We reviewed the literature on BV epidemiology and extracted published data on BV in relation to sexual behaviour and thereby covered published studies and congress proceedings going back for more than half a century. We did not accomplish to unravel BV epidemiology, or to present a definite understanding of disease acquisition. A potential pitfall to our review, is that a considerable number of data reviewed relate to a single pathogen, in particular *G. vaginalis*, involved in a polymicrobial overgrowth condition. Though *G. vaginalis *is a key pathogen to BV, also numerically dominant in the biofilm mode of overgrowth in BV, it is not necessarily the BV initiating or causative agent.

Nonetheless, several conclusions, even if partially supported by circumstantial evidence, can be put forward.

First of all, it is clear that BV differs from established STDs in as much that BV is not confined to women who are, or were, ever engaged in a heterosexual relationship, indicating that, if anything, heterosexual penetrative contact is definitely not a necessary prerequisite to the acquisition of BV.

Among children and prepubertal girls, BV is rather rare, except in case of sexual abuse, even though vaginal carriage of *G. vaginalis *identified through FISH is not uncommon in young girls according to one recent study [26]. Similarly, based on culture-dependent study, *G. vaginalis *seems to be virtually absent in prepubertal boys.

Once beyond the menarche however, BV is observed even among adolescent girls and sexually inexperienced girls, and young women in general, at an appreciable frequency, though on average at significantly lower rates as compared to sexually active adolescents and young women These findings do actually correlate well on what has been observed on the epidemiology of *G. vaginalis *carriage among sexually active versus non-sexually active adolescents [86,87].

Girls and young women who have never engaged in penetrative sexual contact are likely to have practised non-penetrative heterosexual behaviours however, and Tabrizi *et al *documented that among virginal women *G. vaginalis *carriage was very strongly associated with oral sex and non-penetrative digito-genital contact [75]. Similarly, Fethers *et al *very recently documented an association among 17-21-year-old females between noncoital sexual practices (oral sex and non-penetrative digito-genital contact) and the occurrence of BV [76].

These studies are further in line with several other studies that have identified oral receptive sex as a risk factor for BV [67-69]. An obvious critique would be that these studies suffer from confounding, however Schwebke *et al *for instance performed a thorough multivariable analysis, thereby controlling for a number of factors, and found that only receptive oral sex retained significance as a risk factor to unstable vaginal microflora, indicating microflora shifting away from *Lactobacillus *dominance to a BV-like profile [69].

So overall, epidemiological data obtained from adolescent girls and young women, point at an alternative pathogenesis model, not observed with traditionally defined STDs, this is, while the evidence corroborates on the one hand the sexual nature of BV, it clearly also contradicts at least to some extent a traditional infectious disease-like route of disease acquisition.

Furthermore, there is convincing evidence ensuing from condom studies [63,64], from studies among heterosexual versus homosexual men [30], and from studies among young, monogamous men [65,66], that transmission of BV-associated micro-organisms may occur, however that female-to-male transmission may be a far more common route than male-to-female transmission. This sheds a completely different light on the high concordance rates of *G. vaginalis *carriage among couples of whom the female partner has BV, which has been a longstanding argument in favour of BV as an STD.

Data on BV epidemiology among WSW in turn support both aforementioned pathogenetic pathways, i.e. the high rates of BV among WSW may point either at female-to-female route of transmission, either at sexual enhancement of BV occurrence through non-coital sexual behaviours, similar to what is observed among heterosexual couples. Indeed, it has been observed that WSW not only present rather consistently with very high prevalence rates of *G. vaginalis *and bacterial vaginosis, but also that monogamous lesbian couples present with high concordance rates of vaginal microflora characteristics in general. This was recently confirmed by Marrazzo *et al *in terms of concordance between *Lactobacillus *species shared by monogamous lesbian couples [92]. It remains elusive how this concordance is established, though likely to be sexually related. Marrazzo *et al *thereby also pointed at a putative disease mechanism. In particular, recent receptive digital-vaginal sex apparently affected the vaginal *Lactobacillus *community composition and specifically, receptive digital-vaginal sex was associated with the presence of *L. gasseri*, whereas the presence of *L. gasseri *in turn was associated with an increased BV risk [92]. The presence of *L. gasseri *(and *L. iners*) was recently also found among heterosexual pregnant women to confer an increased BV risk [93].

So as a first set of conclusions, it appears as if (1) women with BV may transmit BV-associated micro-organisms to their male or female sexual partners and (2) that non-coital sexual behaviours may enhance the occurrence of BV, which may relate to an effect of such non-penetrative heterosexual and homosexual behaviours on the *Lactobacillus *composition of the vaginal niche, or by the introduction of *G. vaginalis *and/or other BV-related anaerobes in the vagina, or by both.

Finally, while there is ample evidence that both protected and unprotected penetrative sexual behaviour is at the very least a risk marker if not a causal factor to BV, it remains to be answered for how sexual contact is related to BV occurrence and whether this involves sexual transmission of BV-related micro-organisms from a male to a female host. Two alternative explanations emerge from the literature however.

Firstly, an interesting observation was published in 1971 by Leppäluoto [94]. Leppäluoto concluded - based on the study of a large series of Papanicolaou smears taken before and after unprotected coitus - that, lactobacilli-dominated precoital smears were replaced by *G. vaginalis *dominant microflora in postcoital smears [94]. Hence, coitus seems to be associated with a temporary disbalance in the delicate vaginal microflora equilibrium in favour of BV-related micro-organisms. Leppäluoto further launched the idea that the temporary imbalance of the vaginal microflora towards a BV-like profile serves sperm survival and transport, and further speculated that this imbalance is actually physiological in nature, if the microflora is allowed enough time to recover following a coital act [94]. This in turn might concur with the observation made by Vallor *et al*, that loss of hydrogen peroxide producing lactobacilli is primarily explained by the frequency of intercourse [95], while a lack or loss of hydrogen peroxide producing lactobacilli is a strong risk factor to the occurrence of BV [55,96-99].

The most straightforward explanation to this coital effect on the vaginal microflora is that unprotected sexual intercourse alters the physico-chemical vaginal environment thereby also affecting the vaginal microflora. In particular, it has been shown that the alkaline prostatic content of the ejaculate raises the vaginal pH, which remains elevated up to eight hours following coitus [100]. The alkaline ejaculate neutralizes the vaginal pH and the reacidificiation rate of the vagina is estimated (based on *in vitro *experiments) to proceed at about 0.75 pH units per hour in the presence of log8 lactobacilli [101]. Anaerobes grow preferably at a higher pH. The optimum pH for *G. vaginalis *is 6 to 6.5 [102] and it is 5 for *P. bivia *[103]. Hence, unprotected coitus most likely induces an imbalance at the level of the vaginal growth conditions and epithelial binding sites in favour of BV-associated micro-organisms as consistently shown in a large series of postcoital smears by Leppäluoto [94]. Hence, increased coital activity may be one of the reasons why lactobacilli are losing the plot [104].

However, if this were the only mechanism involved than condom use would be expected to act as a very effective means of prevention - which it is not - and hence the proposed mechanism involving a pH-increase does not quite explain why BV acquisition is almost equally enhanced by protected intercourse, as outlined above. Possibly, condom use might be protective against incident BV, but this effect may be obscured in epidemiological studies due to recurrent BV in a substantial number of women, not prevented by condom use.

As a second alternative mechanism, apart from the coital pH-mediated effect on the vaginal microflora, another conceivable mechanism that may be considered is that vaginal penetration somehow promotes the transfer of perianal, perineal, and perivulvar bacteria to the vagina, thereby possibly inducing BV in some women. A similar mechanism has been observed for urinary tract infections in women, and in particular, a significantly elevated risk for urinary tract infection with *E. coli *associated with condom use has been observed [105], presumably involving a rectal-vestibular-urethral pathway. A most important piece of evidence to this postulate with regard to BV, comes from a study by Eschenbach *et al *in which they aimed to document the effects of a single episode of intercourse on the vaginal microflora in subjects randomly assigned to groups that used no condom or lubricated (nonspermicide) condoms [106]. It was found that the 22 subjects who used no condoms had significantly more *E. coli *at a high concentration (> 10^5 ^cfu/mL) in the vagina following unprotected intercourse. However, among the 20 subjects who did use condoms there was also a marginally significant trend towards more vaginal *E. coli *(p = 0.06) and a highly significant increase in other enteric gram-negative rods (p = 0.001) after protected intercourse [106]. It is therefore conceivable that also other, non-coital sexual acts such as oral sex endanger the vaginal microflora through the transfer of BV-associated bacteria from the rectal and perineal regions to the vulvar region and the vagina in analogy to what has been observed for urinary tract infections [107].

Taken together, the abovementioned data suggest that unprotected intercourse may affect the vaginal microflora status through a suppressive effect on lactobacillary colonisation, on the colonisation resistance of the H_2_O_2_-producing lactobacilli, and through the introduction of enteric gram-negative bacteria, but equally, that even protected intercourse is associated with a significant increase of enteric bacteria in the vagina. These mechanisms therefore suggest that BV may behave as a *sexually enhanced disease *rather than an (exclusively) *sexually transmitted infection*. A critical factor - as outlined above - thereby presumably is the frequency of intercourse as has been shown for BV [95] and for urinary tract infection [108,109], possibly by not granting the vaginal ecosystem to restore after a coital act.

Finally, it may be concluded that at present, there is little evidence in support of some kind of male-to-female sexual transmission of BV-associated pathogens. It may be acknowledged however that this transmission route cannot be ruled out based on the available evidence. First, with the surge of molecular studies of the vaginal microflora, a whole new series of previously unknown bacteria has been identified with bacterial vaginosis [110,111] and it is not known whether these species might be transferred through sexual contact and whether these may act as BV-inducing micro-organisms. Secondly, there are some epidemiological data that cannot be explained solely by the sexually enhanced disease model, in particular the observation in the Rakai study [62] that male circumcision is strongly protective against BV acquisition, possibly pointing at the presence of BV-associated bacteria on the prepuce, as recently corroborated by Swidsinski *et al *[26].

In summary, while there is inconclusive evidence in support of male-to-female sexual transmission, we propose that the strong association between sexual behaviour - and level of sexual activity in particular - may be explained by an alternative pathogenetic model, which implies that BV might behave as a *sexually enhanced disease *rather than a *sexually transmitted infection*. This model is in principle consistent with most epidemiological observations cited in this review.

## Summary

• *G. vaginalis *carriage and BV occurs rarely with children, but is common among adolescent, even sexually non-experienced girls, contradicting that sexual transmission a is necessary prerequisite to disease acquisition.

• *G. vaginalis *carriage is enhanced by penetrative sexual contact but also by non-penetrative digito-genital contact and oral sex, again indicating that sex *per se*, but not necessarily coital transmission is involved.

• Several observations also point at female-to-male rather than at male-to-female transmission of *G. vaginalis*, presumably explaining high concordance rates of *G. vaginalis *carriage among couples. Male antibiotic treatment has not been found to protect against BV, condom use is slightly protective, whereas male circumcision might protect against BV.

• BV is also common among women-who-have-sex-with-women and this relates at least in part to non-coital sexual behaviours.

• Though male-to-female transmission cannot be ruled out, overall there is little evidence that BV acts as an STD. Rather, BV may be considered a sexually enhanced disease, with frequency of intercourse being a critical factor.

## Competing interests

The authors declare that they have no competing interests.

## Authors' contributions

HV drafted the manuscript, MV made substantial revisions of the draft, RV and MT provided important intellectual content that added to the manuscript.

All authors read and approved the final version of the manuscript.

## Pre-publication history

The pre-publication history for this paper can be accessed here:

http://www.biomedcentral.com/1471-2334/10/81/prepub
